# Odds Ratio Estimation of Medical Students’ Attitudes towards COVID-19 Vaccination

**DOI:** 10.3390/ijerph18136815

**Published:** 2021-06-25

**Authors:** Miroslava Sovicova, Jana Zibolenova, Viera Svihrova, Henrieta Hudeckova

**Affiliations:** Department of Public Health, Jessenius Faculty of Medicine in Martin, Comenius University in Bratislava, Mala Hora 11149/4B, 036 01 Martin, Slovakia; miroslava.sovicova@uniba.sk (M.S.); jana.zibolenova@uniba.sk (J.Z.); henrieta.hudeckova@uniba.sk (H.H.)

**Keywords:** COVID-19, vaccination, medical students

## Abstract

This study investigated the attitudes of Slovak medical students to COVID-19 vaccination. A cross-sectional study was conducted between 10 March 2021 and 24 March 2021, as the second wave of coronavirus spread in Slovakia. It was performed in four medical faculties with students in years 1–6. An online anonymous questionnaire was distributed through official university platforms. The survey was completed by 1228 of 5374 medical students. The vaccinated group of students had received at least one dose of the COVID-19 vaccine. The study was conducted on 1228 students, of which 880 (71.7%) were vaccinated and 348 unvaccinated (28.3%). The median age was 22 years (range 18–33 years), and 70.6% were women. The lowest vaccination rate was among first (32.7%) and second-year students (61.6%), students living at home with their family (63.8%) and students living in urban areas (69.8%). Only 22.4% of medical students were concerned about serious side effects from the COVID-19 vaccine, and 38.8% were concerned that the COVID-19 vaccine may not be effective. This study provides key information related to medical student vaccination in Slovakia and education about COVID-19 vaccination.

## 1. Introduction

The COVID-19 pandemic is the third major outbreak in twenty years of respiratory disease related to the coronavirus. There have been more than 153 million confirmed cases of COVID-19, including 3 million deaths globally so far [1]. The first cases of coronavirus infection in Slovakia were reported on 6 March 2020. Thanks to quick response and strict preventive measures, the country became a leader in the fight against the first wave of the coronavirus pandemic. Unfortunately, in the second wave, the new cases of coronavirus rose rapidly, and the country became one of the most affected in Europe [2]. An increasing number of workers succumbed to coronavirus infections, and there was a problem with workforce shortage in hospitals. To prevent the healthcare system from being overwhelmed, Slovakia, as well as other European countries, decided to involve medical students as volunteers or to stage their practical learning in hospitals [3].

At the end of 2020, the new hope to counter the global pandemic—the COVID-19 vaccine—appeared. The first vaccines against COVID-19 were administered to the general public in Slovakia on 5 January [4]. Healthcare workers are frequently exposed to infected individuals with COVID-19 while providing healthcare, which places makes them at higher risk of infection than others [5], so the country prioritized the vaccination of healthcare workers and medical students who were in contact with COVID-19 patients. At present, Slovakia is using all of the COVID-19 vaccines approved by the European Medicines Agency [6,7].

The vaccination can be successful only given high rates of acceptance and coverage [8]. The attitudes of medical students should have a high level of acceptance for the vaccination according to their attitude to vaccination, as well as to protect their health and the health of patients from COVID-19 [9,10]. Health professionals were identified as the most important source of information on vaccination for the general public [11,12], and previous studies have shown that medical students play an important role in vaccination too [9,13]. Vaccinated students with a positive attitude towards vaccines and sharing their vaccination experiences with patients can encourage vaccine intake [9]. There is thus a need to investigate the overall attitudes towards COVID-19 vaccination in medical students to plan an effective vaccination strategy for healthcare workers and patient protection [9,10,13,14].

The aim of our study was to investigate the attitudes of Slovak medical students to COVID-19 vaccination.

## 2. Materials and Methods

### 2.1. Participants and Procedure

A cross-sectional study was conducted between 10 March 2021 and 24 March 2021, in the period just after the peak of the second wave of the coronavirus spread in Slovakia. The study was conducted in all four medical faculties from three universities in Slovakia—Jessenius Faculty of Medicine in Martin (JFM CU) and Faculty of Medicine of Comenius University in Bratislava (FM CU), Faculty of Medicine, Pavol Jozef Safarik University in Kosice (FM UPJS) and Faculty of Medicine, Slovak Medical University in Bratislava (FM SZU). Our target population included students of general medicine in the 1st through 6th years from these faculties.

**Figure 1:**
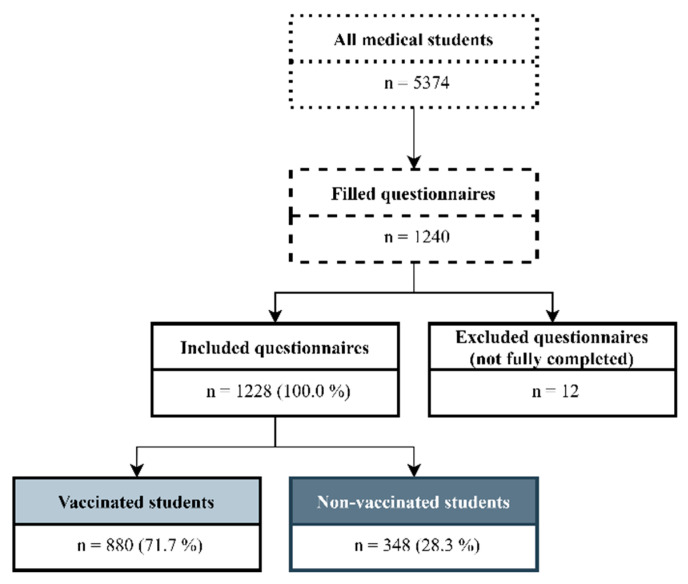
Study flow chart.

An online anonymous questionnaire was hosted in Microsoft Forms and distributed through official university platforms on social media (e.g., Facebook). The survey was completed by 1240 of 5374 medical students from all four faculties of medicine in Slovakia [15]. Twelve respondents were excluded due to missing data or being foreign students, so the final sample size was 1228 students. The students who had received at least one dose of the COVID-19 vaccine until the time of data collection were included in the vaccinated group (*N* = 880). The students who had not received any vaccine were included in the unvaccinated group (*N* = 348) (Figure 1).

### 2.2. Study Instrument

The questionnaire was adopted from authors Lucia et al. with their consent [9]. Several questions were changed and added because of the timing of the study and the regional context. The questionnaire consisted of the following sections: demography, general attitudes to the vaccine, attitudes to the COVID-19 disease and the COVID-19 vaccine, personal experience with COVID-19, and personal vaccination behaviour specially focused on flu and the COVID-19 vaccine. Most of the questions were assigned as mandatory-to-answer items to avoid incompleteness and missing data. It took, on average, 8 min to complete the questionnaire.

### 2.3. Statistical Analyses

Data were extracted from Microsoft Forms to an Excel spreadsheet and then statistically analysed using Epi Info 7 and R software, version 4.0.2 [16,17]. Categorical variables were expressed as frequencies and percentages. Responses (attitudes) that were recorded on a four-point Likert scale (strongly agree, agree, disagree, strongly disagree) were split into two categories: “agree” and “disagree”. The association between categorical variables was analysed using the chi-squared test of independence. Multivariate logistic regression was used to identify predictors of COVID-19 vaccination status. Odds ratios with 95% confidence intervals were used to identify statistically significant differences between the vaccinated and unvaccinated groups. Statistically significant differences were on the level of 5% (*p* < 0.05).

## 3. Results

The study was conducted on 1228 students, of which 880 (71.7%) were vaccinated and 348 unvaccinated (28.3%). The median student age was 22 years (range 18–33 years), and 70.6% were female. Other socio-demographic characteristics of the sample are shown in Table 1.

Significant differences were identified between vaccination status and faculty, year of study and usual residence. The lowest vaccination rate was for first- and second-year students, whilst the vaccination rate at higher school years was statistically significantly higher. Respondents living at home with their families were less likely to be vaccinated compared to respondents who lived on the campus or on their own in a private residence. Respondents living in an urban area were less likely to be vaccinated than respondents living in a rural area, but the difference was not significant (Table 1).

Respondents who have not had a COVID-19 infection or worked as a medical volunteer during the pandemic were more likely to get a COVID-19 vaccine. Delay in vaccination and the decision to not get the vaccine as an adult for other reasons than illness or allergy were associated with a lower COVID-19 vaccination rate. Surprisingly, routine vaccination against flu was not significantly associated with a higher COVID-19 vaccination rate, but getting a flu vaccine in the last flu season (2020/2021) was associated with a higher COVID-19 vaccination rate (Table 2).

Almost all of the measured attitudes (except “It is my role as a future physician to learn about vaccines for myself and my patients”) were significantly associated with COVID-19 vaccination status in univariate analysis. Generally, the attitudes of most medical students towards COVID-19 vaccination are compliant with vaccination; only 22.4% of medical students were concerned about serious side effects from the COVID-19 vaccine, and 38.8% were concerned that a COVID-19 vaccine might not be effective (Table 2).

**Figure 2:**
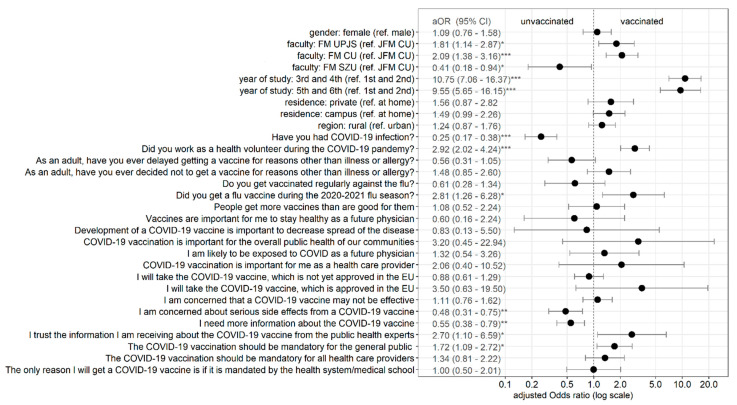
Predictors of COVID-19 vaccination status among medical students—multivariate logistic regression analysis. Note: * *p* < 0.05; ** *p* < 0.01; *** *p* < 0.001; Reference category in attitudes questions is not affirmative (no or disagree + strongly disagree).

Results of multivariate logistic regression with COVID-19 vaccination status as the dependent variable are shown in Figure 2. All variables that were significantly associated with vaccination status at the 0.1 level in the univariate analysis (chi-squared test) were included in the model. The model was also controlled for insignificant factors—gender and routine vaccination against flu. School years (grades) were merged into couples. Of demographic predictors, being a 3rd and 4th school-year student or a 5th and 6th school-year student was highly associated with being more likely to get the COVID-19 vaccine in comparison with being a 1st and 2nd school-year student. Significant differences in vaccination rates among different faculties of medicine were also observed. Having a COVID-19 infection was associated with a lower probability of getting the COVID-19 vaccine, whilst working as a medical volunteer during the pandemic and a previous flu vaccination in season 2020/2021 were associated with a higher probability of getting the COVID-19 vaccine. Those concerned about serious side effects from a COVID-19 vaccine and needing more information about the COVID-19 vaccine were less likely to get the COVID-19 vaccine. Those who trusted the information received from public health experts and who thought the COVID-19 vaccination should be mandatory for the general public were more likely to get the COVID-19 vaccine.

## 4. Discussion

Healthcare professionals are at increased risk of COVID-19 infection. In the absence of protective equipment, this risk can be significantly increased. This situation can subsequently cause the collapse of the health care system during a COVID-19 pandemic [18].

While the vaccination looks promising in the fight against COVID-19, attitudes towards the COVID-19 vaccine have become a challenge for health professionals. As physicians play an important role in influencing vaccination decisions, medical students can be helpful in vaccine advocacy in the general public, too [9,12,13]. The latest studies on COVID-19 vaccination focus mainly on the acceptance of this new vaccine among medical students. Vaccine acceptance varies across the time, place and perceived behavioural nature of the community [19,20]. For instance, a study in Italy immediately after the lifting of the lock-down (June–July) observed positive attitudes towards vaccination among more than 94% of students [21]. Surprisingly, a similar study on willingness to be vaccinated among American medical students from September 2020 obtained only a 60.6% acceptance rate [22]. In our study, 96.1% of Slovak medical students had a positive attitude towards vaccination.

Although a recent study on factors affecting attitudes towards the new COVID-19 vaccine in Slovenian medical students showed male (66% vs. 55%) students as more compliant in taking the COVID-19 vaccine [23], we did not observe any statistically significant difference in acceptance by gender (male 72.6% vs. 71.3% female). On the other hand, we did identify differences in vaccination status within the faculties and year of study. The medical students from different universities and cities showed different levels of vaccination, and the gaps between vaccinated students within the different universities could depend on information about opportunities to be vaccinated and its interpretation. According to the Slovak COVID-19 vaccination strategy, only medical students in contact with a patient can receive the vaccine. In consideration of the curriculum of Slovak medical students, this means that students in the 3–6 years of study could be registered for vaccination.

The Slovak public health authority accepted in their COVID-19 vaccine strategy they recommended 90 days as the duration of immunological memory after infection [6]. Therefore, individuals in this period who were at the beginning of vaccination were recommended to delay receiving the vaccine. This could explain why the respondents in our study who had already had a COVID-19 infection were less likely to get the COVID-19 vaccine than those who had never been infected.

Although most studies confirmed the impact of seasonal flu vaccination on COVID-19 vaccine acceptance [21,24], this was not found in our results. However, only 8.8% of Slovak medical students are regularly vaccinated against the seasonal flu, although 13.8% of the Slovak medical students who were vaccinated against the flu in the last season also obtained a COVID-19 vaccination. Because influenza can increase the mortality rate and hospitalizations—especially during the COVID-19 pandemic—flu vaccination represents an effective public health mitigation strategy for the pandemic [21].

Our study confirmed that 93.6% of all Slovak medical students trusted the information about the COVID-19 vaccine received from public health experts, compared to 98.2% of vaccinated Slovak medical students. A similar finding was reported in the study by Kekelar et al., where 87% of students mentioned public health experts as an important source of information about the COVID-19 vaccine [14]. Qiao et al. introduced confidence in scientists, as well as health agencies, as the strongest predictors for acceptance of the vaccine against COVID-19 among American college students (83.1% of students trusted in scientists, 70.2% in health agencies) [21]. Interestingly, 56.3% of Slovak medical students agreed that the COVID-19 vaccination should be mandatory for the general public; this attitude towards the importance of the vaccine was also found among Egyptian medical students (69.7%) and medical students from Michigan (67.9%) [9,10].

Vaccine hesitancy is specific to one’s environment and fluctuates according to time, setting, and the vaccines themselves. There are many factors that influence hesitancy, ranging from doubt or complete refusal [10,20,25,26]. In our study, 28.3% of medical students were not vaccinated. More than 96.8% of medical students in the Egyptian study by Saied et al. were concerned about the adverse effects of the COVID-19 vaccine, and 93.2% of all respondents were concerned about its effectiveness [10]. Likewise, the study by Lucia et al. found that more than 54% of American medical students were concerned about serious side-effects of the COVID-19 vaccine, and 76.7% of all respondents were concerned about its ineffectiveness [9,10,14]. Of our respondents, only 22.4% were concerned about serious side-effects and 38.8% had a problem with its ineffectiveness.

### Limitations and Strengths

One limitation of our study was not comparing the attitudes of Slovak medical students with healthcare professionals who had the same vaccination options and the same information about the vaccine, but with more medical experience.

There are several studies on medical students’ attitudes towards COVID-19 vaccination, each using a country-specific questionnaire. The questionnaires are not standardized, which could lead to problems in interpreting the internationals’ results.

At present, there are not enough studies on the vaccination of medical students against COVID-19, and therefore the strength of this study is the new knowledge about the attitudes of Slovak medical students to vaccination.

## 5. Conclusions

After several months of an ongoing vaccination program in Slovakia, our study showed that most Slovak medical students accepted COVID-19 vaccination; moreover, most of them obtained the vaccine. It is necessary to focus on the correct interpretation of the vaccination strategy for medical students. An appropriate education curriculum designed to enhance student knowledge about the COVID-19 vaccine will be important in the fight against the COVID-19 pandemic.

## Figures and Tables

**Table 1:** Demographic factors associated with vaccination status among medical students in Slovakia.

		All Respondents (*N* = 1228) *N* (%)	Vaccinated Group (*N* = 880) *N* (%)	Unvaccinated Group (*N* = 348) *N* (%)	*p* Value
gender	male	361 (29.4)	262 (72.6)	99 (27.4)	0.646
	female	867 (70.6)	618 (71.3)	249 (28.7)	
faculty	JFM CU	461 (37.5)	326 (70.7)	135 (29.3)	0.027
	FM CU	454 (37.0)	346 (76.2)	108 (23.8)	
	FM SZU	53 (4.3)	34 (64.2)	19 (35.8)	
	FM UPJS	260 (21.2)	174 (66.9)	86 (33.1)	
year of study	1	263 (21.4)	86 (32.7)	177 (67.3)	<0.0001
	2	185 (15.1)	113 (61.6)	72 (38.9)	
	3	285 (23.2)	238 (83.5)	47 (16.5)	
	4	201 (16.4)	184 (91.5)	17 (8.5)	
	5	184 (15.0)	163 (88.6)	21 (11.4)	
	6	110 (9.0)	96 (87.3)	14 (12.7)	
residence	home	279 (22.7)	178 (63.8)	101 (36.2)	0.004
	campus	788 (64.2)	581 (73.7)	207 (26.3)	
	private	161 (13.1)	121 (75.2)	40 (24.8)	
region	urban	771 (62.8)	538 (69.8)	233 (30.2)	0.057
	rural	457 (37.2)	342 (74.8)	115 (25.2)	

**Table 2:** Survey responses among COVID-19 vaccinated and unvaccinated groups. Univariate analysis.

Survey Item	All Respondents (*N* = 1228) *N* (%)	Vaccinated Group (*N* = 880) *N* (%)	Unvaccinated Group (*N* = 348) *N* (%)	*p* Value
Experience with COVID-19	Participants that responded affirmatively (yes)			
Have you had COVID-19 infection?	250 (20.4)	137 (15.6)	113 (32.5)	<0.0001
Do you personally know someone who has had COVID-19 infection?	1216 (99.0)	873 (99.2)	343 (98.6)	0.303
Did you work as a health volunteer during the COVID-19 pandemic?	623 (50.7)	532 (60.5)	91 (26.1)	<0.0001
Do you personally know someone who has died from COVID-19 infection?	578 (47.1)	413 (46.9)	165 (47.4)	0.879
Personal vaccination behavior				
As an adult, have you ever delayed getting a vaccine for reasons other than illness or allergy?	172 (14.0)	108 (12.3)	64 (18.4)	0.005
As an adult, have you ever decided not to get a vaccine for reasons other than illness or allergy?	227 (18.5)	144 (16.4)	83 (23.9)	0.002
Do you get vaccinated regularly against the flu?	108 (8.8)	82 (9.3)	26 (7.5)	0.303
Did you get flu vaccine during the 2020–2021 flu season?	141 (11.5)	121 (13.8)	20 (5.7)	<0.0001
General attitudes to vaccine	Participants that responded affirmatively (agree/strongly agree)			
People get more vaccines than are good for them	88 (7.2)	44 (5.0)	44 (12.6)	<0.0001
Vaccines are important for me to stay healthy as a future physician	1191 (97.0)	870 (98.9)	321 (92.2)	<0.0001
It is my role as a future physician to learn about vaccines for myself and my patients	1221 (99.4)	876 (99.5)	345 (99.1)	0.393
Attitudes to COVID-19 disease and COVID-19 vaccine				
Development of a COVID-19 vaccine is important to decrease spread of the disease	1200 (97.7)	877 (99.7)	323 (92.8)	<0.0001
COVID-19 vaccination is important for the overall public health of our communities	1196 (97.4)	878 (99.8)	318 (91.4)	<0.0001
I am likely to be exposed to COVID as a future physician	1187 (96.7)	862 (98.0)	325 (93.4)	<0.0001
COVID-19 vaccination is important for me as a health care provider	1173 (95.5)	875 (99.4)	298 (85.6)	<0.0001
I will take the COVID-19 vaccine, which is not yet approved in the EU	291 (23.7)	193 (21.9)	98 (28.2)	0.021
I will take the COVID-19 vaccine, which is approved in the EU	1180 (96.1)	877 (99.7)	303 (87.1)	<0.0001
I am concerned that a COVID-19 vaccine may not be effective	476 (38.8)	306 (34.8)	170 (48.9)	<0.0001
I am concerned about serious side effects from a COVID-19 vaccine	275 (22.4)	125 (14.2)	150 (43.1)	<0.0001
I need more information about the COVID-19 vaccine	599 (48.8)	367 (41.7)	232 (66.7)	<0.0001
I trust the information I am receiving about the COVID-19 vaccine from the public health experts	1150 (93.6)	864 (98.2)	286 (82.2)	<0.0001
The COVID-19 vaccination should be mandatory for the general public	691 (56.3)	562 (63.9)	129 (37.1)	<0.0001
The COVID-19 vaccination should be mandatory for all health care providers	880 (71.7)	689 (78.3)	191 (54.9)	<0.0001
The only reason I will get a COVID-19 vaccine is if it is mandated by the health system/medical school	88 (7.2)	32 (3.6)	56 (16.1)	<0.0001

## Data Availability

All data are fully available without any restriction upon reasonable request.
